# Comparison of agar-based methodologies to broth disc elution for the detection of aztreonam susceptibility in the presence of ceftazidime-avibactam

**DOI:** 10.1128/spectrum.03794-25

**Published:** 2026-03-10

**Authors:** Teslin S. Sandstrom, Andra Banete, Alice Kanyua, Kevin M. Davis, Susan M. Poutanen

**Affiliations:** 1 Department of Laboratory Medicine and Pathobiology, Temerty Faculty of Medicine, University of Toronto 233837, Toronto, Ontario, Canada; 2 Temerty Faculty of Medicine, University of Toronto 12366, Toronto, Ontario, Canada; 3 Department of Pathology and Laboratory Medicine, Aga Khan University 58585 , Nairobi, Kenya; 4 Department of Microbiology, University Health Network/Sinai Health 7989, Toronto, Ontario, Canada; Universita degli Studi dell'Insubria, Varese, Italy

**Keywords:** aztreonam-avibactam, metallo-beta-lactamase, multi-drug resistant, broth disc elution, validation, Enterobacterales

## Abstract

The combination of aztreonam and ceftazidime-avibactam is a proven treatment option for infections caused by metallo-beta-lactamase (MBL)-expressing gram-negative organisms. Restoration of aztreonam susceptibility in the presence of ceftazidime-avibactam can be determined using the Clinical and Laboratory Standards Institute (CLSI)’s broth disc elution (BDE) method; however, the high manual workload and significant material footprint of this assay make it difficult to implement in a high-throughput clinical microbiology lab. This study evaluates two agar-based methods for the detection of susceptibility to the combination of aztreonam and ceftazidime-avibactam, using BDE as the comparator methodology. Resistant Enterobacterales, *Pseudomonas aeruginosa*, *Stenotrophomonas maltophilia*, and *Acinetobacter* spp. were selected, after which restoration of aztreonam susceptibility was tested using an E-test/disc diffusion (E-DD) method, a double-disc diffusion (DDD) method, and BDE. In comparison to BDE, both E-DD and DDD methods met CLSI M52 validation thresholds with respective categorical agreement rates of 93% and 96%, and no major (MDs) or very major discrepancies (VMDs). In conclusion, agar-based methods such as E-DD or DDD provide alternative methods to evaluate restoration of aztreonam susceptibility in the presence of ceftazidime-avibactam, which can subsequently be used to inform patient care decisions.

**IMPORTANCE:**

Infections caused by multi-drug-resistant, gram-negative organisms with limited treatment options are associated with significant morbidity and mortality. To avoid patient harm caused by delays in appropriate antimicrobial therapy, the clinical microbiology laboratory must be able to test and report susceptibility to novel antimicrobials or combinations of antimicrobials. The combination of aztreonam plus ceftazidime-avibactam is an empiric, first-line regimen for metallo-beta-lactamase-producing gram-negative organisms that has historically been used without prior susceptibility testing due to a lack of access to gold-standard methods. The Clinical and Laboratory Standards Institute published a broth-based method to test this combination, but it is challenging to implement in a busy clinical laboratory. Two easier-to-implement agar-based methods were successfully validated, which are more accessible and less resource-intensive than the currently described broth-based assays.

## INTRODUCTION

Carbapenemase-producing gram-negative organisms are recognized as a global health threat and a World Health Organization (WHO) priority pathogen group (1–3). Included within this are metallo-beta-lactamase (MBL)-expressing Enterobacterales, which are resistant to common beta-lactam/beta-lactamase inhibitor combinations and frequently exhibit reduced susceptibility to monobactam antibiotics such as aztreonam due to the co-expression of Class A carbapenemases and extended-spectrum beta-lactamases (ESBLs) (4).

The Infectious Disease Society of America (IDSA) recommends either cefiderocol (expensive and difficult to access in Canadian healthcare settings) or a combination of aztreonam and ceftazidime-avibactam as first-line therapies for MBL-expressing Enterobacterales (5). The latter is estimated to be effective against approximately 90% of MBL-expressing Enterobacterales (5, 6) and relies on avibactam to “shield” aztreonam from hydrolysis by Ambler Class A and D serine carbapenemases, as well as Class C ESBLs (7). Subsequently, the resistance of aztreonam to hydrolysis by Class B MBLs is leveraged to achieve antimicrobial activity.

Ceftazidime-avibactam and aztreonam are also recommended for *Stenotrophomonas maltophilia*, with avibactam protecting aztreonam from hydrolysis by its native L2 serine beta-lactamase (5). Although the combination of aztreonam and ceftazidime-avibactam is not recommended for the treatment of infections caused by organisms other than Enterobacterales and *S. maltophilia,* a small proportion of MBL-expressing *Pseudomonas aeruginosa* are susceptible to this combination (8, 9). Limited data suggest a reduction in aztreonam minimum inhibitory concentration (MIC) in the presence of ceftazidime-avibactam for *Acinetobacter* spp. (10–12).

The implementation of a reliable, low-barrier assay for evaluation of restored aztreonam susceptibility in the presence of ceftazidime-avibactam has direct patient care implications. *In lieu* of resource-intensive gold-standard methods such as broth microdilution (BMD) or agar dilution, more simplistic means by which to determine susceptibility have been proposed using agar-based testing. These include gradient diffusion strip stacking or crossing, disc stacking, double disc diffusion, E-test/disc diffusion (E-DD), or antibiotic-supplemented agar (13–17). However, results are variable, and none of these methods have been standardized. The Clinical and Laboratory Standards Institute (CLSI) has proposed the broth disc elution (BDE) method (18), which has been validated against gold-standard methodologies and uses supplies that are readily accessible in clinical laboratories. While more accessible than BMD, the BDE method is difficult to perform in a high-throughput clinical microbiology laboratory due to high workload and sizable material footprint.

The purpose of this study was to validate two agar-based methods using BDE as the comparator methodology. The E-test/disc diffusion (E-DD) (15) and double-disc diffusion (DDD) (13) methods were chosen due to their use of readily available laboratory materials and technical simplicity. To reduce subjectivity of assay interpretation, CLSI zone measurement breakpoints for aztreonam were used to confirm susceptibility or resistance in the presence of ceftazidime-avibactam. A secondary objective was to document the prevalence of susceptibility to the combination of ceftazidime-avibactam and aztreonam among resistant Enterobacterales, *S. maltophilia*, *P. aeruginosa*, and *Acinetobacter* spp.

## MATERIALS AND METHODS

### Study design

This validation study was designed in accordance with CLSI M52 Ed. 1: Verification of Commercial Microbial Identification and Antimicrobial Susceptibility Testing Systems criteria (19). For the purposes of this evaluation, the BDE assay, which is a CLSI-endorsed methodology used to assess restoration of aztreonam susceptibility in the presence of ceftazidime-avibactam, was used as the comparator (i.e., reference) method. Antimicrobial susceptibility testing using E-DD and DDD was performed in parallel with BDE using the same standard inoculum prepared from 24-h sub-cultures of clinical isolates and quality control organisms (described in detail below). Reading and interpretation of assay results was performed by three independent readers. For reads that required measurement of zone size, the average of the three independent measurements was used. E-DD and DDD methods were considered successfully validated when categorical agreement (CA) was ≥90%, very major discrepancies (VMDs) were <3%, major discrepancies (MDs) were <3%, and minor discrepancies (MinD) were ≤10%. As stipulated by the CLSI M52, the term “discrepancy” has been used rather than “error” because a non-gold-standard method was used as a comparator method. Because BDE does not provide a range of MIC values, essential agreement (EA) was not evaluated. All isolates that demonstrated either a VMD or MD following the initial round of testing were subsequently re-tested by all three methods, in triplicate, using new sub-cultures. These results were again read and interpreted by three independent readers. The result that reflected the mode of the repeat testing interpretations was used following recommendations by Humphries et al. (20).

Because there are currently no established CLSI breakpoints for aztreonam-avibactam, zone measurement breakpoints for aztreonam (for Enterobacterales and *P. aeruginosa*) were used to establish a read of “susceptible,” “intermediate,” or “resistant” for organisms tested by E-DD or DDD. *P. aeruginosa* breakpoints were also used for *S. maltophilia* and *Acinetobacter* spp., as these organisms do not have established CLSI zone measurement breakpoints for aztreonam.

### Reagents

Antibiotics used in this study included 30/20 µg ceftazidime-avibactam discs (Liofilchem, Italy), 30 µg aztreonam discs (Oxoid Ltd., Thermo Fisher Scientific, Waltham, MA, USA), ceftazidime-avibactam antibiotic gradient strips (E-test , bioMérieux, France), and 10 µg meropenem discs (Oxoid Ltd., Thermo Fisher Scientific). Cation-adjusted Mueller-Hinton broth (CA-MHB) 5 mL tubes, 5% sheep blood agar plates, and Mueller-Hinton agar plates were purchased from Thermo Fisher Scientific. Standardized quality control organisms *Escherichia coli* 25922, *Klebsiella pneumoniae* BAA-1705, and *K. pneumoniae* BAA-2146 were obtained from the American Type Culture Collection (ATCC).

### Clinical isolates

A total of 94 isolates with previously measured resistance to aztreonam and ceftazidime-avibactam were included in this validation study. These included 35 Enterobacterales, 19 *S. maltophilia,* 23 *P. aeruginosa*, and 17 *Acinetobacter* spp. isolated from clinical samples and patient surveillance swabs collected from patients admitted to four academic acute care hospitals in Toronto, Canada between 2014 and 2024. All isolates were stored at −80°C and were sub-cultured onto 5% sheep blood agar with antibiotic pressure (10 µg meropenem disc placed in main inoculum) prior to further antimicrobial susceptibility testing. For all Enterobacterales isolates, carbapenemase genes detected by the Xpert Carba-R PCR (Cepheid) were recorded using historical information available within the laboratory information system (LIS; SCC Soft Computer). Carba-R PCR was not repeated following subculture; however, all organisms included in this validation study maintained resistance to ceftazidime-avibactam and aztreonam in isolation, as confirmed by the broth disc elution method (further discussed below and depicted in Table S1). Carbapenemase gene expression for other isolates was only available and recorded if the isolate had previously been characterized by the Canadian National Microbiology Laboratory.

### Quality control organisms

Three ATCC organisms (*E. coli* ATCC 25922, *K. pneumoniae* BAA-1705, and *K. pneumoniae* BAA-2146) were included with each experimental run to ensure appropriate quality control, and for reproducibility testing purposes. As per CLSI M52 criteria for reproducibility testing (19), two clinical isolates (one fully resistant *P. aeruginosa* and one *S*. *maltophilia* that demonstrated susceptibility to aztreonam in the presence of avibactam) were selected for inclusion with each experimental run. These isolates were selected from a panel of organisms evaluated during proof-of-concept testing of the BDE method.

### Broth disc elution

BDE was performed as outlined by Harris et al. (18) and the CLSI M100 Ed. 34, Table 3D (21). Briefly, three CA-MHB tubes were inoculated with their respective antibiotic disc(s)—aztreonam, ceftazidime-avibactam, or aztreonam plus ceftazidime-avibactam—and allowed to incubate at room temperature for 30 min. An additional fourth tube was reserved as a growth control with no antibiotic added. A 0.5 McFarland standard inoculum was prepared, and 25 μL of this was inoculated into each broth tube yielding a final concentration of approximately 7.5 × 10^5^ CFU/mL. The standard inoculum was also used to inoculate a purity plate, as well as two Mueller-Hinton agar plates for additional testing (described below). Broth tubes were incubated at 33–35°C, ambient air for 16–20 h and subsequently examined visually for evidence of turbidity, which was reported as bacterial growth. For the purpose of using consistent language when comparing BDE to E-DD and DDD methods, isolates for which turbidity was seen (and would therefore be considered “non-susceptible” by the CLSI M100 standards) were called “resistant.” Isolates for which no turbidity was seen were called “susceptible.”

### E-test/disc diffusion

E-DD was performed as described by Rawson et al. (15). A 0.5 McFarland standard inoculum was used to inoculate a lawn of organism onto a Mueller-Hinton agar plate, onto which a ceftazidime-avibactam E-test strip and an aztreonam disc were placed 15 mm (center to center), with the aztreonam disc aligned with the MIC breakpoint for both Enterobacterales and *P. aeruginosa* (8 μg/mL). This setup was also used for clinical isolates without established ceftazidime-avibactam clinical breakpoints. Plates were incubated at 33–35°C, ambient air for 16–20 h and subsequently examined for evidence of restored susceptibility to aztreonam in the presence of ceftazidime-avibactam by two methods, as depicted in Fig. 2a. First, plates were visually examined for an enlarged zone of clearance between the ceftazidime-avibactam E-test strip and the aztreonam disc. For the purposes of comparison to BDE, the presence of any zone, regardless of size or shape, was deemed to be evidence of restored aztreonam susceptibility (and was recorded as “susceptible”), provided it originated from the aztreonam disc and extended toward the E-test strip. Isolates which had a zone of clearance that did not meet this description or had no zone visualized were recorded as “resistant.” Next, as previously described by Rawson et al. (15), the radius of the zone of clearance between E-test strip and disc was measured in duplicate from center of aztreonam disc to the outer edge of the zone. An average of these measurements was then multiplied by two to obtain a final zone diameter. This measurement step was performed for all isolates, regardless of the size or shape of the observed zone of clearance. Zone diameters were interpreted as susceptible, intermediate, or resistant as per CLSI M100 aztreonam breakpoints for Enterobacterales or *P. aeruginosa. P. aeruginosa* breakpoints were also used for *Acinetobacter* spp. and *S. maltophilia* as CLSI zone measurement breakpoints for these organisms are not established.

### Double disc diffusion

DDD was performed as described by Falcone et al. (13) and Verschelden et al. (16). A 0.5 McFarland standard inoculum was used to inoculate a lawn of organism onto a Mueller-Hinton agar plate, onto which aztreonam and ceftazidime-avibactam discs were placed 20 mm apart (center to center). Plates were incubated at 33–35°C, ambient air for 16–20 h and subsequently examined for evidence of restored susceptibility to aztreonam in the presence of ceftazidime-avibactam in the same manner as described above for the E-DD assay (Fig. 3a).

### Precision testing

Intra-run variability was assessed by calculating precision categorical agreement (PCA) for each of the five quality control organisms, for each methodology, over the course of three independent runs carried out over three sequential weeks. Precision was considered verified when >95% of the results were within PCA.

### Statistics

95% confidence intervals (CIs) for point estimates were calculated manually via the modified Wald method, using GraphPad QuickCalcs software.

### Artwork

**Fig 1:**
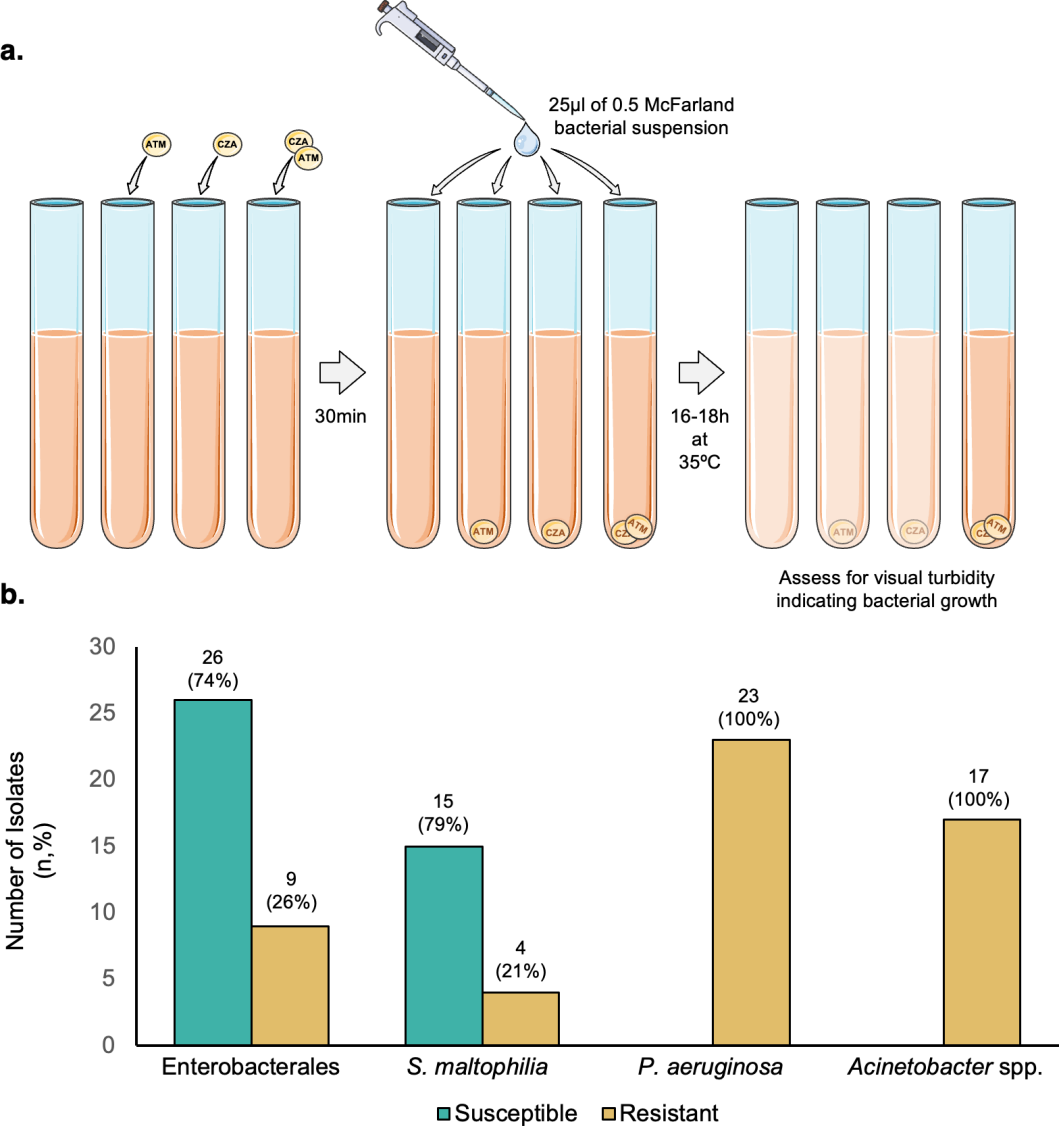
Restoration of aztreonam susceptibility in the presence of ceftazidime-avibactam can be detected by broth disc elution. (**a**) Experimental set up of BDE assay. (**b**) Frequency of susceptible (teal) and resistant (gold) isolates within each organism sub-group, as detected by BDE. Total number of organisms is denoted by inlay number within each column.

**Fig 2:**
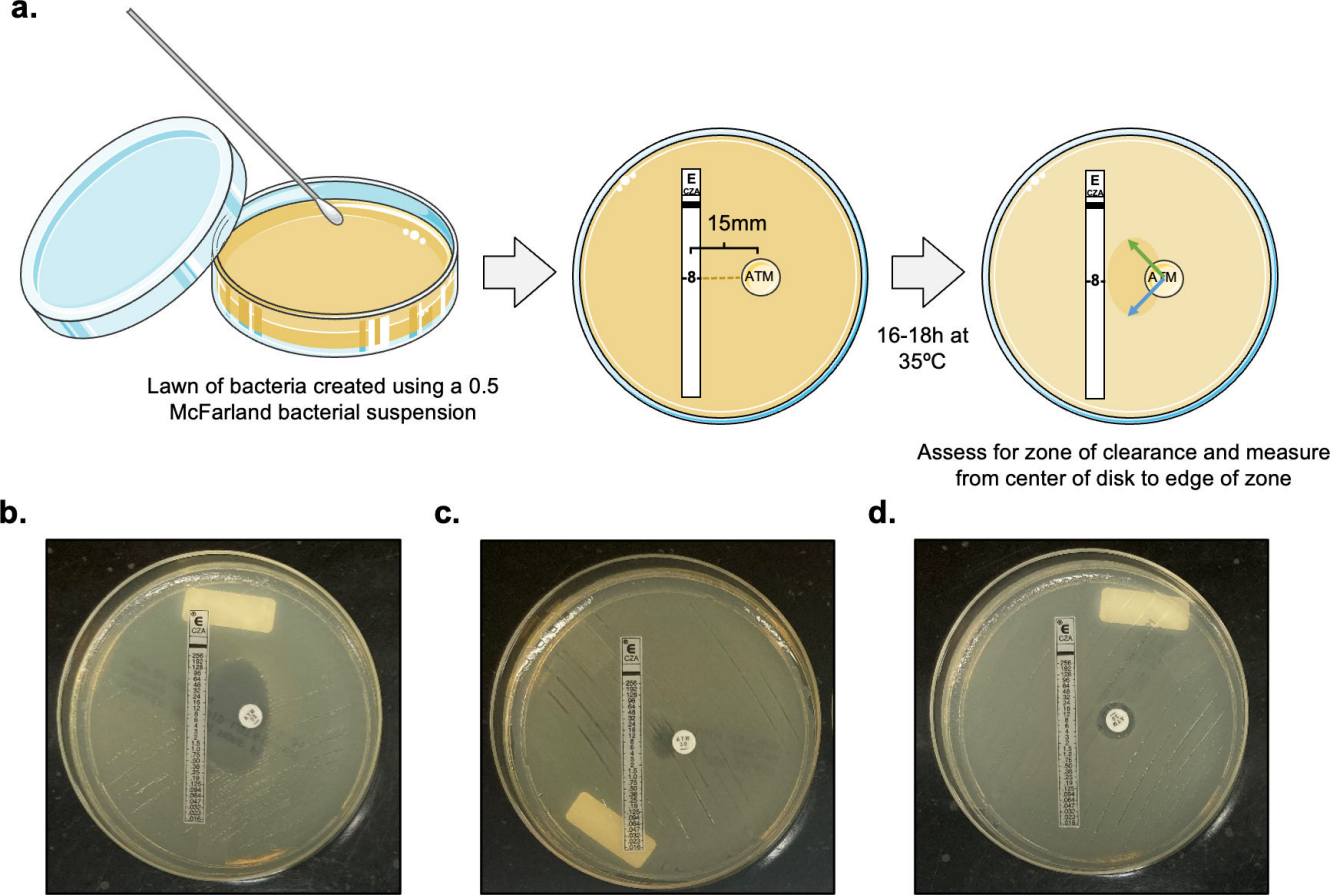
Restoration of aztreonam susceptibility in the presence of ceftazidime-avibactam can be detected by E-test/disc diffusion. (**a**) Experimental set-up of E-DD assay. Duplicate measurements of the resulting zone of clearance, as depicted by blue and green arrows, were used to obtain an average zone diameter measurement that was then compared to CLSI zone measurement breakpoints for aztreonam. (**b–d**) depict various plate appearances. (**b**) Example of an isolate with a large zone of clearance, with calculated aztreonam zone diameter corresponding to an interpretation of “susceptible.” (**c**) Example of an isolate with a small zone of clearance, with calculated aztreonam zone diameter corresponding to an interpretation of “resistant.” (**d**) Example of an isolate exhibiting no increase in size of aztreonam zone diameter.

**Fig 3:**
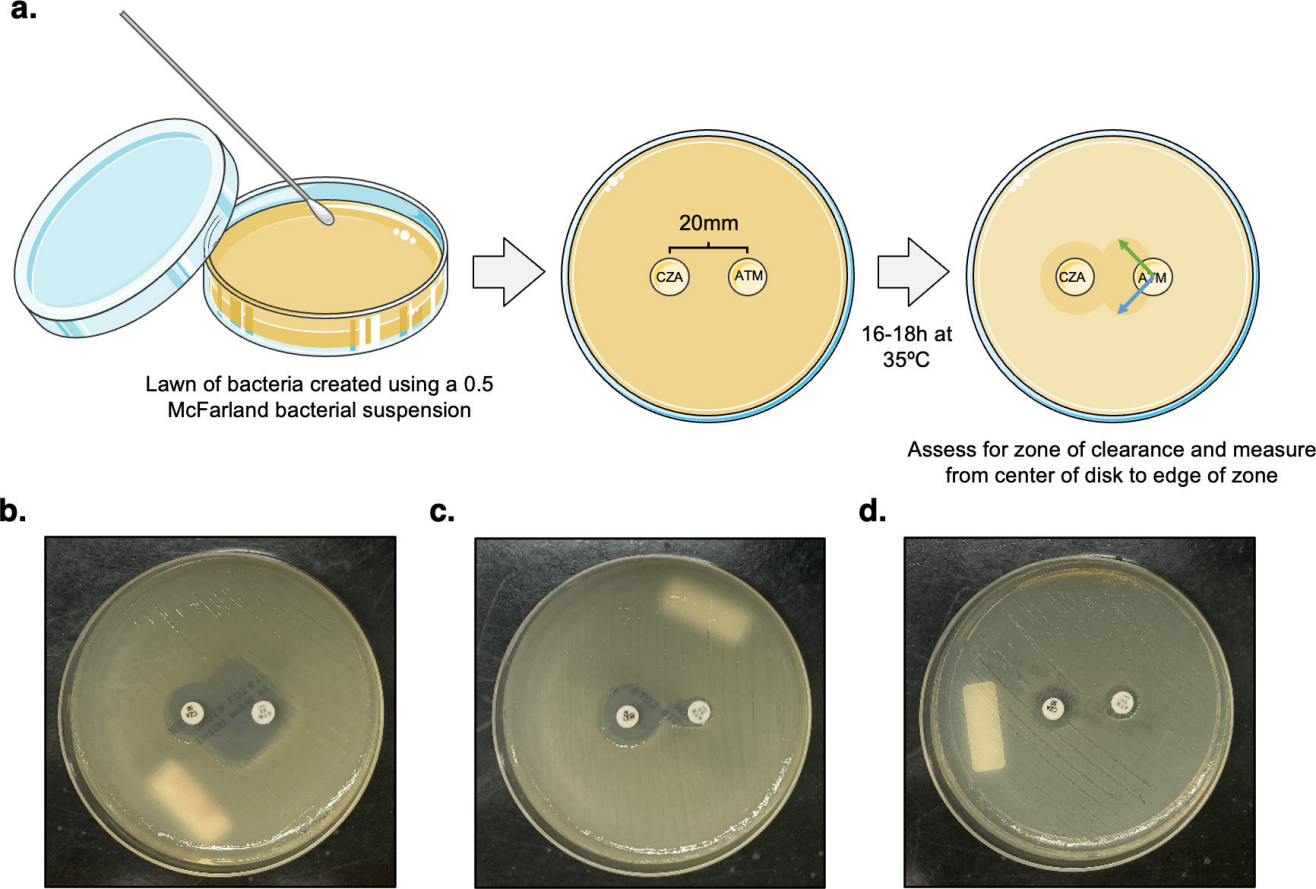
Restoration of aztreonam susceptibility in the presence of ceftazidime-avibactam can be detected by double disc diffusion. (**a**) Experimental set-up of DDD assay. Duplicate measurements of the resulting zone of clearance, as depicted by blue and green arrows, were used to obtain an average zone diameter measurement that was then compared to CLSI zone measurement breakpoints for aztreonam. (**b–d**) depict various plate appearances. (**b**) Example of an isolate with a large zone of clearance, with calculated aztreonam zone diameter corresponding to an interpretation of “susceptible.” (**c**) Example of an isolate with a small zone of clearance, with calculated aztreonam zone diameter corresponding to an interpretation of “resistant.” (**d**) Example of an isolate exhibiting no increase in size of aztreonam zone diameter.

Figures 1a, 2a, and 3a were created using Smart Servier Medical Art image sets (available from https://smart.servier.com/). Servier Medical Art by Servier is licensed under CC BY 4.0.

## RESULTS

### Isolate characteristics

A total of 94 unique bacterial isolates from clinical samples and patient surveillance swabs (i.e., routine rectal swabs collected on admitted patients to inform infection prevention and control practices) collected between 2014 and 2024 met study inclusion criteria. These included Enterobacterales (*n* = 35), *S. maltophilia* (*n* = 19), *P. aeruginosa* (*n* = 23), and *Acinetobacter* spp. (*n* = 17). Isolates and mechanisms of antimicrobial resistance obtained from the laboratory information system data (if available from prior testing) are listed in Table S1. Represented MBLs included New Delhi metallo-beta-lactamase (NDM), Verona Integron–encoded (VIM), and imipenemase (IMP) metallo-beta-lactamases.

### Broth disc elution allows detection of restored aztreonam susceptibility in the presence of ceftazidime-avibactam

Using BDE (Fig. 1a), 26 out of 35 (74% [95% CI, 58–86]) Enterobacterales and 15 out of 19 (79% [95% CI, 56–92]) *S. maltophilia* demonstrated restored aztreonam susceptibility in the presence of ceftazidime-avibactam (Fig. 1b). Resistant isolates among the Enterobacterales sub-group were predominantly *E. coli* (*n* = 8), with one *K. pneumoniae* isolate also demonstrating growth in the aztreonam plus ceftazidime-avibactam broth tube. All *P. aeruginosa* and *Acinetobacter* spp. isolates were resistant to aztreonam in the presence of ceftazidime-avibactam.

### Validation of E-test/disc diffusion to assess restoration of aztreonam susceptibility in the presence of ceftazidime-avibactam

Restoration of aztreonam susceptibility in the presence of ceftazidime-avibactam was first assessed by E-DD, as depicted in Fig. 2a. Results were subsequently compared to BDE to calculate the CA, VMD, MD, and MinD for this method.

As shown in Table 1, the CA of the E-DD method in comparison to BDE was 96% (95% CI, 89–99) when interpreted by visualization alone, with a VMD rate of 8% (95% CI, 3–18) and no MDs. MinDs were not calculated as visualization alone did not allow for an interpretation of “intermediate” to be applied to either the BDE or E-DD method. The VMD rate of 8% was explained by 4 isolates (all *E. coli*) that were resistant to aztreonam plus ceftazidime-avibactam by BDE but also demonstrated a very small zone of clearance interpreted by the study criteria as restored aztreonam susceptibility. For clarity, representative examples of various zone appearances are shown in Fig. 2b through d, with panel 2c demonstrating the small zone of clearance seen in one such isolate. Panel 2c is representative of the small zone of clearance seen for all four isolates that were associated with a VMD.

**TABLE 1:** Comparison of E-DD and BDE methods, using visualization of zone of clearance to interpret restoration of aztreonam susceptibility in the presence of ceftazidime-avibactam.

Agreement or discrepancy category	Frequency (%) (95% CI)				
	Total (*n* = 94)	Enterobacterales (*n* = 35)	*S. maltophilia* (*n* = 19)	*P. aeruginosa* (*n* = 23)	*Acinetobacter* spp. (*n* = 17)
CA	96 (89–99)	89 (74–96)	100 (80–100)	100 (83–100)	100 (78–100)
VMD	8 (3–18)	44 (19–73)	0 (0–55)	0 (0–17)	0 (0–22)
MD	0 (0–10)	0 (0–15)	0 (0–24)	N.D.^*a*^	N.D.^*a*^

^*a*^ N.D. = not determined; unable to calculate value due to lack of sensitive isolates within this sub-group.

Comparatively, using aztreonam zone diameter breakpoints to determine susceptibility versus resistance resulted in a CA rate of 93% (95% CI, 85–97) and a MinD rate of 7% (95% CI, 3–15) (Table 2). The previously observed VMDs were resolved by this interpretation method, since zone diameter measurements for all four isolates were interpreted using CLSI M100 breakpoints as either intermediate (*n* = 2) or resistant (*n* = 2). MinDs were accounted for by seven isolates, with no specific trends in directionality (i.e., whether they overcalled resistance [MD] or susceptibility [VMD] in comparison to the comparator method). The 2 Enterobacterales and 1 *P. aeruginosa* isolate demonstrated a result of “resistant” by reference BDE and therefore trended toward a VMD, whereas the 4 *S. maltophilia* isolates had a result of “susceptible” by reference BDE and therefore trended toward an MD.

**TABLE 2:** Comparison of E-DD and BDE methods, using calculated zone diameter and corresponding CLSI zone measurement breakpoints for aztreonam to interpret restoration of aztreonam susceptibility in the presence of ceftazidime-avibactam.

Agreement or discrepancy category	Frequency (%) (95% CI)				
	Total (*n* = 94)	Enterobacterales (*n* = 35)	*S. maltophilia* (*n* = 19)	*P. aeruginosa* (*n* = 23)	*Acinetobacter* spp. (*n* = 17)
CA	93 (85–97)	94 (80–99)	79 (56–92)	96 (77–100)	100 (78–100)
VMD	0 (0–8)	0 (0–35)	0 (0–55)	0 (0–17)	0 (0–22)
MD	0 (0–10)	0 (0–15)	0 (0–24)	N.D.^*a*^	N.D.^*a*^
MinD	7 (3–15)	6 (1–20)	21 (8–44)	4 (0–23)	0 (0–22)

^*a*^ N.D. = not determined; unable to calculate value due to lack of sensitive isolates within this sub-group.

Next, data were re-analyzed by organism subset. The CA, VMD, MD, and MinD rates for the Enterobacterales met CLSI M52 validation criteria when using zone measurement breakpoints as part of the interpretation strategy (Table 2). While CA, VMD, and MinD rates were acceptable for *P. aeruginosa* and *Acinetobacter* spp., there were no isolates that were susceptible to aztreonam plus ceftazidime avibactam by the comparator method, and therefore the MD rate was not calculated. Conversely, the *S. maltophilia* subset demonstrated a CA rate of 79% (95% CI, 56–92), with a MinD rate of 21% (95% CI, 8–44) and no VMDs or MDs.

Finally, because a result of “intermediate” is often interpreted by clinical teams to be analogous to “resistant,” data were re-analyzed by considering isolates that had intermediate aztreonam zone measurements to be resistant for the purposes of comparison to BDE. As shown in Table S2, this resulted in a CA of 96% (95% CI, 89–99), but an increased MD rate of 10% (95% CI, 3–23).

### Validation of double-disc diffusion to assess restoration of aztreonam susceptibility in the presence of ceftazidime-avibactam

Restoration of aztreonam susceptibility in the presence of ceftazidime-avibactam was next assessed by the DDD method, as depicted in Fig. 3a, using the same interpretation strategies outlined above. Results were subsequently compared to BDE to calculate CA, VMD, MD, and MinD for this assay.

As shown in Table 3, the CA of the DDD method in comparison to BDE was 93% (95% CI, 85–97) when interpreted by visualization alone, with a VMD rate of 13% (95% CI, 6–25), and no MDs. MinDs were not calculated as visualization did not allow for an interpretation of “intermediate” to be applied to either the BDE or DDD method. As was observed for the E-DD method, the VMD rate of 13% for DDD was explained by 7 isolates that were resistant to aztreonam plus ceftazidime-avibactam by BDE but also demonstrated a very small zone of clearance interpreted by study criteria as restored aztreonam susceptibility. This is shown in Fig. 3b through d, with panel 3c demonstrating the small zone of clearance seen in one such isolate. Zone diameter measurements for all 7 isolates were interpreted using CLSI M100 breakpoints as either intermediate (*n* = 3) or resistant (*n* = 4).

**TABLE 3:** Comparison of DDD and BDE methods, using visualization of zone of clearance to interpret restoration of aztreonam susceptibility in the presence of ceftazidime-avibactam.

Agreement or discrepancy category	Frequency (%) (95% CI)				
	Total (*n* = 94)	Enterobacterales (*n* = 35)	*S. maltophilia* (*n* = 19)	*P. aeruginosa* (*n* = 23)	*Acinetobacter* spp. (*n* = 17)
CA	93 (85–97)	89 (74–96)	89 (67–98)	96 (77–100)	100 (78–100)
VMD	13 (6–25)	44 (19–73)	50 (15– 85)	4 (0–23)	0 (0–22)
MD	0 (0–10)	0 (0–15)	0 (0–24)	N.D.^*a*^	N.D.^*a*^

^*a*^ N.D. = not determined; unable to calculate value due to lack of sensitive isolates within this sub-group.

When interpretation was performed by visualization of zone of clearance followed by measurement of zone diameter with application of CLSI aztreonam breakpoints, this methodology yielded a CA rate of 96% (95% CI, 89–99) and a MinD rate of 4% (95% CI, 1–11) with no VMDs or MDs (Table 4). MinDs for DDD trended toward VMDs and were attributed to 1 Enterobacterales isolate, 1 *P. aeruginosa* isolate, and 2 *S*. *maltophilia* isolates that demonstrated a result of “resistant” by BDE.

**TABLE 4:** Comparison of DDD and BDE methods, using calculated zone diameter and corresponding CLSI zone measurement breakpoints for aztreonam to interpret restoration of aztreonam susceptibility in the presence of ceftazidime-avibactam.

Agreement or discrepancy category	Frequency (%), (95% CI)				
	Total (*n* = 94)	Enterobacterales (*n* = 35)	*S. maltophilia* (*n* = 19)	*P. aeruginosa* (*n* = 23)	*Acinetobacter* spp. (*n* = 17)
CA	96 (89–90)	97 (84–100)	89 (67–98)	96 (77–100)	100 (78–100)
VMD	0 (0–8)	0 (0–35)	0 (0–55)	0 (0–17)	0 (0–22)
MD	0 (0–10)	0 (0–15)	0 (0–24)	N.D.^*a*^	N.D.^*a*^
MinD	4 (1–11)	3 (0–16)	11 (2–33)	4 (0–23)	0 (0–22)

^*a*^ N.D. = not determined; unable to calculate value due to lack of sensitive isolates within this sub-group.

Similarly to E-DD, CA, VMD, MD, and MinD rates for the Enterobacterales met CLSI M52 validation criteria when applying zone measurement breakpoints (Table 4). CA, VMD, and MinD rates were again acceptable for *P. aeruginosa* and *Acinetobacter* spp., but without isolates that tested as susceptible to aztreonam plus ceftazidime avibactam by the comparator method, the MD rate for these subsets could not be calculated. The *S. maltophilia* subset demonstrated a CA rate of 89% (95% CI, 67–98), with a MinD rate of 11% (95% CI, 2–33) and no VMDs or MDs.

As was done for the E-DD method comparison, the DDD results were re-analyzed by considering isolates that had intermediate aztreonam zone measurements to be resistant for the purposes of comparison to BDE. As shown in Table S3, this resulted in a CA of 100% (95% CI, 89–99) with no VMDs or MDs.

### Precision testing

PCA was calculated using results from the five previously designated experimental control organisms (3 ATCC organisms and 2 clinical isolates). These were run weekly over three sequential weeks, the results of which were read and interpreted by at least three independent reviewers. PCA for each organism was 100% for BDE, DDD, and E-DD.

## DISCUSSION

In the last decade, infections caused by carbapenemase-producing organisms, especially those with MBLs, have increased in frequency in Canadian acute care centers (4). This has prompted requests for antimicrobial susceptibility testing of newer antimicrobials, including the combination of aztreonam plus ceftazidime-avibactam. Testing this combination using the CLSI M100 BDE method is challenging. This project validated two simpler methods—E-DD and DDD—against BDE as the comparator method.

In this validation study, E-DD and DDD were interpreted by qualitative and quantitative measurement methods. Quantitative assessment (measuring the zone of clearance) performed better than qualitative testing and allowed both methods to meet the CLSI M52 threshold standards for assay validation. The lack of accuracy of qualitative testing has been shown before; agar-based methods for the evaluation of aztreonam susceptibility in the presence of ceftazidime-avibactam often lack standardized methods for interpretation, which results in discordance when being validated against BMD (17). Because of these challenges, a zone measurement step for both E-DD (as per previously published literature [15]) and DDD methods was used. In doing so, all VMDs observed for both qualitative agar-based methods were resolved. Notably, isolates that yielded VMDs produced very small zones of clearance that were visually different than the large zones of clearance observed with aztreonam/ceftazidime-avibactam susceptible isolates. Nonetheless, a measurement step removes subjectivity, ensures objective interpretation when training new users, and can facilitate accurate comparison should serial testing be performed.

In addition to informing laboratory practices, this study provided an estimate of the prevalence of local isolates that exhibit susceptibility to the combination of aztreonam and ceftazidime-avibactam. For MBL-expressing Enterobacterales, 74% (95% CI, 58–86) of isolates were susceptible in comparison to the 85–99% predicted by prior literature (6, 8, 9, 22). It is beyond the scope of this work to perform additional analyses to investigate the underlying mechanism(s) of this observation; however, penicillin binding protein 3 (PBP3) mutations, efflux pump alterations, and acquisition of additional beta-lactamase variants that are resistant to avibactam inhibition have been attributed to aztreonam/ceftazidime-avibactam resistance in other studies (23–27). The proportion of *S. maltophilia* isolates susceptible to the combination of ceftazidime-avibactam and aztreonam was also lower than prior estimates of >90% (28), although the small number of isolates included in this study limits this comparison. In contrast to restored aztreonam susceptibility described in a small set of *P. aeruginosa* isolates in a prior large-scale surveillance study published by Biedenbach et al. (8), the current study showed no evidence of the same for any of the *P. aeruginosa* isolates. Biedenbach et al. also demonstrated almost ubiquitous resistance to aztreonam in *Acinetobacter* spp. isolates in the presence of avibactam (8), which is in alignment with the current study findings. These data provide a baseline upon which the prevalence of non-susceptibility to ceftazidime-avibactam and aztreonam can be compared when reviewing organisms that meet study inclusion criteria.

This study has notable limitations. First, the comparator method used was the CLSI BDE method and not the gold-standard BMD. Given that BDE compared well to BMD during initial validation (18), it was felt that using it as a comparator method was an acceptable and pragmatic choice. Second, there was a limited number of clinical isolates within each organism subset, which prevented precise estimates of CA, VMDs, MDs, and MinDs for each specific organism type. This was particularly evident for the *S. maltophilia* isolates, which demonstrated a CA rate of 79% and 89% for E-DD and DDD, respectively, with MinD rates exceeding 10% in both instances. With only 19 isolates included in this subset, the MinD rate of 11% for DDD was attributable to only 2 organisms. The inclusion of a 95% confidence interval when displaying these results was intended to help address this ambiguity. Finally, this study did not evaluate Mueller Hinton broth, Mueller Hinton agar, and antimicrobial discs from different manufacturers. These are materials that are used routinely in our center and as such are purchased from a single manufacturer. Manufacturer variability has been identified as an issue in similar validation studies (18) and thus should be considered if other institutes plan to implement a similar methodology. Reassuringly, lot-to-lot variability for any of the materials used during testing was not seen, as demonstrated by the PCA of 100% for each of the 5 quality control organisms.

### Conclusion

Both E-DD and DDD are useful methods by which to assess susceptibility to the combination of ceftazidime-avibactam and aztreonam, and perform well when compared to BDE. By employing a standardized approach to interpret restored susceptibility to aztreonam (i.e., use of CLSI zone measurement breakpoints), inconsistencies in interpretation that have previously been identified as a major limitation to this style of assay were minimized. Finally, aztreonam-avibactam has been approved by the Federal Drug Agency in the United States of America but is not yet available in Canadian healthcare settings. It is expected that CLSI breakpoints will be released and direct testing with aztreonam-avibactam will become available. In the interim, E-DD and DDD can be utilized for the informed use of aztreonam plus ceftazidime-avibactam for patients with infections caused by multi-drug-resistant gram-negative organisms.

## SUPPLEMENTAL MATERIAL

Supplemental tables spectrum.03794-25-s0001.pdf Tables S1 to S3.

ASM does not own the copyrights to Supplemental Material that may be linked to, or accessed through, an article. The authors have granted ASM a non-exclusive, world-wide license to publish the Supplemental Material files. Please contact the corresponding author directly for reuse.
