# Supplementary material for: Comparison of agar-based methodologies to broth disc elution for the detection of aztreonam susceptibility in the presence of ceftazidime-avibactam
Source: Microbiol Spectr. 2026 Mar 10;14(4):e03794-25. doi: 10.1128/spectrum.03794-25 (PMC13055259; doi:10.1128/spectrum.03794-25)
Supplement: Supplemental tables — Tables S1 to S3. [file spectrum.03794-25-s0001.pdf]

Supplementary Table 1: Organism distribution and carbapenemase expression

Study ID	Organism	Carbapenemase(s) Expression	Beta-lactamase(s) Expression	BDE Results (Growth = "+"; No growth = "-")				E-Test/Disk Diffusion Assay (Yes = "+"; No = "-")			Double-Disk Diffusion Assay (Yes = "+"; No = "-")		
				ATM BDE Result	CZA BDE Result	ATM-CZA BDE Result	ATM-CZA BDE Interpretation	Visualized zone	Average zone diameter (mm)	Interpretation (S/I/R)	Visualized zone	Average zone diameter (mm)	Interpretation (S/I/R)
GNCP7	*Klebsiella pneumoniae*	NDM	*N.D.*	+	+	-	S	+	33	S	+	33	S
GNCP10	*Escherichia coli*	NDM	*N.D.*	+	+	-	S	+	22	S	+	23	S
GNCP15	*Escherichia coli*	NDM	*N.D.*	+	+	+	R	+	20	I	+	19	I
E14	*Klebsiella pneumoniae*	NDM	*N.D.*	+	+	-	S	+	27	S	+	27	S
ENT2	*Escherichia coli*	*N.D.*	CTX-M	+	+	+	R	-	6	R	-	6	R
ENT4	*Escherichia coli*	NDM	*N.D.*	+	+	-	S	+	24	S	+	23	S
ENT5	*Klebsiella pneumoniae*	NDM	*N.D.*	+	+	-	S	+	27	S	+	27	S
ENT6	*Klebsiella pneumoniae*	NDM, OXA-48	*N.D.*	+	+	-	S	+	33	S	+	31	S
ENT7	*Klebsiella pneumoniae*	NDM, OXA-48	*N.D.*	+	+	-	S	+	28	S	+	28	S
ENT8	*Escherichia coli*	NDM, OXA-48	*N.D.*	+	+	+	R	+	14	R	+	16	R
ENT9	*Escherichia coli*	NDM, OXA-48	*N.D.*	+	+	+	R	+	15	R	+	16	R
ENT10	*Escherichia coli*	NDM	*N.D.*	+	+	-	S	+	24	S	+	23	S
ENT12	*Escherichia coli*	NDM	*N.D.*	+	+	-	S	+	35	S	+	35	S
ENT13	*Escherichia coli*	NDM	*N.D.*	+	+	+	R	+	18	I	+	17	R
ENT16	*Escherichia coli*	NDM	OXA-1	+	+	-	S	+	23	S	+	24	S
ENT17	*Escherichia coli*	VIM	SHV	+	+	-	S	+	33	S	+	31	S
ENT18	*Escherichia coli*	NDM	*N.D.*	+	+	+	R	-	9	R	-	10	R
ENT20	*Escherichia coli*	NDM	TEM, CTX-M, OXA-1	+	+	-	S	+	26	S	+	26	S
ENT22	*Escherichia coli*	NDM	*N.D.*	+	+	+	R	-	6	R	-	6	R
ENT23	*Klebsiella pneumoniae*	VIM	SHV, TEM, CTX-M, CMY-2	+	+	+	R	-	6	R	-	6	R
ENT25	*Klebsiella pneumoniae*	NDM, OXA-48	SHV, TEM, CTX-M	+	+	-	S	+	27	S	+	26	S
ENT26	*Escherichia coli*	NDM	*N.D.*	+	+	+	R	-	15	R	-	16	R
ENT27	*Klebsiella pneumoniae*	NDM, OXA-48	*N.D.*	+	+	-	S	+	29	S	+	29	S
ENT28	*Klebsiella pneumoniae*	NDM	*N.D.*	+	+	-	S	+	35	S	+	33	S
ENT29	*Enterobacter cloacae complex*	NDM	*N.D.*	+	+	-	S	+	33	S	+	31	S
ENT31	*Enterobacter cloacae complex*	NDM	*N.D.*	+	+	-	S	+	31	S	+	33	S
ENT36	*Citrobacter amalonaticus*	VIM	*N.D.*	+	+	-	S	+	36	S	+	36	S
ENT37	*Enterobacter cloacae complex*	VIM	*N.D.*	+	+	-	S	+	33	S	+	34	S
ENT38	*Enterobacter cloacae complex*	NDM	*N.D.*	+	+	-	S	+	34	S	+	34	S
ENT41	*Enterobacter cloacae complex*	NDM	*N.D.*	+	+	-	S	+	32	S	+	32	S
ENT42	*Enterobacter cloacae complex*	NDM	*N.D.*	+	+	-	S	+	35	S	+	33	S
ENT43	*Enterobacter hormaechei*	NDM	*N.D.*	+	+	-	S	+	37	S	+	38	S
ENT45	*Enterobacter hormaechei*	VIM	*N.D.*	+	+	-	S	+	33	S	+	33	S
ENT49	*Enterobacter cloacae complex*	VIM	*N.D.*	+	+	-	S	+	37	S	+	36	S
ENT50	*Klebsiella pneumoniae*	NDM	TEM, SHV, CTX-M, OXA-232	+	+	-	S	+	29	S	+	30	S
P5	*Pseudomonas aeruginosa*	*N.A.*	*N.A.*	+	+	+	R	-	19	I	-	19	I
P8	*Pseudomonas aeruginosa*	*N.A.*	*N.A.*	+	+	+	R	-	6	R	-	7	R
P10	*Pseudomonas aeruginosa*	*N.A.*	*N.A.*	+	+	+	R	-	9	R	-	9	R
P24	*Pseudomonas aeruginosa*	*N.A.*	*N.A.*	+	+	+	R	-	12	R	-	13	R
PSA2	*Pseudomonas aeruginosa*	*N.A.*	*N.A.*	+	+	+	R	-	6	R	-	6	R
PSA3	*Pseudomonas aeruginosa*	*N.A.*	*N.A.*	+	+	+	R	-	11	R	-	11	R
PSA4	*Pseudomonas aeruginosa*	*N.A.*	*N.A.*	+	+	+	R	-	6	R	-	6	R
PSA5	*Pseudomonas aeruginosa*	*N.A.*	*N.A.*	+	+	+	R	-	6	R	-	6	R
PSA6	*Pseudomonas aeruginosa*	*N.A.*	*N.A.*	+	+	+	R	-	6	R	-	6	R
PSA8	*Pseudomonas aeruginosa*	*N.A.*	*N.A.*	+	+	+	R	-	14	R	-	14	R
PSA9	*Pseudomonas aeruginosa*	*N.A.*	*N.A.*	+	+	+	R	-	7	R	-	7	R
PSA10	*Pseudomonas aeruginosa*	*N.A.*	*N.A.*	+	+	+	R	-	6	R	-	6	R
PSA11	*Pseudomonas aeruginosa*	*N.A.*	*N.A.*	+	+	+	R	-	6	R	-	7	R
PSA12	*Pseudomonas aeruginosa*	*N.A.*	*N.A.*	+	+	+	R	-	6	R	-	6	R
PSA13	*Pseudomonas aeruginosa*	*N.A.*	*N.A.*	+	+	+	R	-	6	R	-	6	R
PSA14	*Pseudomonas aeruginosa*	*N.A.*	*N.A.*	+	+	+	R	-	11	R	-	10	R
PSA15	*Pseudomonas aeruginosa*	*N.A.*	*N.A.*	+	+	+	R	-	6	R	-	6	R
PSA16	*Pseudomonas aeruginosa*	*N.A.*	*N.A.*	+	+	+	R	-	6	R	-	6	R
PSA17	*Pseudomonas aeruginosa*	*N.A.*	*N.A.*	+	+	+	R	-	6	R	-	6	R
PSA19	*Pseudomonas aeruginosa*	*N.A.*	*N.A.*	+	+	+	R	-	6	R	-	6	R
PSA22	*Pseudomonas aeruginosa*	*N.A.*	*N.A.*	+	+	+	R	-	6	R	-	6	R
PSA23	*Pseudomonas aeruginosa*	*N.A.*	*N.A.*	+	+	+	R	-	7	R	+	9	R
PSA24	*Pseudomonas aeruginosa*	*N.A.*	*N.A.*	+	+	+	R	-	6	R	-	9	R
GNAS1	*Acinetobacter baumannii complex*	*N.A.*	*N.A.*	+	+	+	R	-	8	R	-	8	R
GNAS3	*Acinetobacter baumannii complex*	*N.A.*	*N.A.*	+	+	+	R	-	13	R	-	14	R
GNAS10	*Acinetobacter baumannii complex*	OXA-51, OXA-23	*N.D.*	+	+	+	R	-	8	R	-	8	R
GNAS14	*Acinetobacter baumannii complex*	OXA-51, OXA-23	*N.D.*	+	+	+	R	-	6	R	-	6	R
GNAS16	*Acinetobacter baumannii complex*	OXA-51, OXA-23	*N.D.*	+	+	+	R	-	10	R	-	11	R
AC3	*Acinetobacter baumannii complex*	OXA-51, OXA-23	*N.D.*	+	+	+	R	-	9	R	-	9	R
AC4	*Acinetobacter baumannii complex*	OXA-51, OXA-23	*N.D.*	+	+	+	R	-	9	R	-	10	R
AC5	*Acinetobacter baumannii complex*	OXA-51, OXA-23	*N.D.*	+	+	+	R	-	6	R	-	6	R
AC6	*Acinetobacter baumannii complex*	OXA-51, OXA-23	*N.D.*	+	+	+	R	-	6	R	-	8	R
AC9	*Acinetobacter baumannii complex*	*N.A.*	*N.A.*	+	+	+	R	-	6	R	-	6	R
AC11	*Acinetobacter baumannii complex*	*N.A.*	*N.A.*	+	+	+	R	-	6	R	-	6	R
AC18	*Acinetobacter baumannii*	*N.A.*	*N.A.*	+	+	+	R	-	12	R	-	13	R
AC19	*Acinetobacter baumannii complex*	*N.A.*	*N.A.*	+	+	+	R	-	14	R	-	15	R
AC20	*Acinetobacter baumannii complex*	*N.A.*	*N.A.*	+	+	+	R	-	10	R	-	10	R
ACN1	*Acinetobacter baumannii*	NDM	*N.D.*	+	+	+	R	-	6	R	-	6	R
ACN2	*Acinetobacter baumannii*	NDM, OXA-51, OXA-23	*N.D.*	+	+	+	R	-	6	R	-	6	R
ACN3	*Acinetobacter baumannii*	OXA-51, OXA-24	*N.D.*	+	+	+	R	-	8	R	-	7	R
GNSM10	*Stenotrophomonas maltophila*	*N.A.*	*N.A.*	+	+	-	S	+	21	I	+	29	S
GNSM2	*Stenotrophomonas maltophila*	*N.A.*	*N.A.*	+	+	+	R	-	6	R	+	18	I
SM1	*Stenotrophomonas maltophila*	*N.A.*	*N.A.*	+	+	-	S	+	24	S	+	28	S
SM12	*Stenotrophomonas maltophila*	*N.A.*	*N.A.*	+	+	-	S	+	28	S	+	26	S
STM1	*Stenotrophomonas maltophila*	*N.A.*	*N.A.*	+	+	-	S	+	20	I	+	30	S
STM2	*Stenotrophomonas maltophila*	*N.A.*	*N.A.*	+	+	-	S	+	32	S	+	38	S
STM3	*Stenotrophomonas maltophila*	*N.A.*	*N.A.*	+	+	-	S	+	28	S	+	29	S
STM9	*Stenotrophomonas maltophila*	*N.A.*	*N.A.*	+	+	+	R	-	6	R	-	6	R
STM10	*Stenotrophomonas maltophila*	*N.A.*	*N.A.*	+	+	-	S	+	22	S	+	28	S
STM12	*Stenotrophomonas maltophila*	*N.A.*	*N.A.*	+	+	-	S	+	31	S	+	32	S
STM13	*Stenotrophomonas maltophila*	*N.A.*	*N.A.*	+	+	-	S	+	26	S	+	27	S
STM14	*Stenotrophomonas maltophila*	*N.A.*	*N.A.*	+	+	-	S	+	24	S	+	29	S
STM15	*Stenotrophomonas maltophila*	*N.A.*	*N.A.*	+	+	+	R	-	6	R	-	6	R
STM18	*Stenotrophomonas maltophila*	*N.A.*	*N.A.*	+	+	+	R	-	7	R	+	20	I
STM20	*Stenotrophomonas maltophila*	*N.A.*	*N.A.*	+	+	-	S	+	26	S	+	31	S
STM21	*Stenotrophomonas maltophila*	*N.A.*	*N.A.*	+	+	-	S	+	32	S	+	32	S
STM23	*Stenotrophomonas maltophila*	*N.A.*	*N.A.*	+	+	-	S	+	21	I	+	28	S
STM24	*Stenotrophomonas maltophila*	*N.A.*	*N.A.*	+	+	-	S	+	26	S	+	28	S
STM25	*Stenotrophomonas maltophila*	*N.A.*	*N.A.*	+	+	-	S	+	20	I	+	24	S

N.D. = Not detected; N.A. = Not assessed; S = susceptible; I = Intermediate; R = Resistant

Supplementary Table 2: Performance of E-test/disc diffusion assay by measurement

Agreement or Discrepancy Category	Frequency (%), (95% CI)				
	Total (n=94)	Enterobacterales (n=35)	*S. maltophilia* (n=19)	*P. aeruginosa* (n=23)	*Acinetobacter* spp. (n=17)
CA	96 (89–99)	100 (88–100)	79 (56–92)	100 (83–100)	100 (78–100)
VMD	0 (0–8)	0 (0–35)	0 (0–54)	0 (0–17)	0 (0–22)
MD	10 (3–23)	0 (0–15)	27 (11–52)	N.D.*	N.D.*

*N.D. = Not determined; unable to calculate value due to lack of sensitive isolates within this sub-group

Supplementary Table 3: Performance of double-disc diffusion assay by measurement

Agreement or Discrepancy Category	Frequency (%), (95% CI)				
	Total (n=94)	Enterobacterales (n=35)	*S. maltophilia* (n=19)	*P. aeruginosa* (n=23)	*Acinetobacter* spp. (n=17)
CA	100 (95–100)	100 (88–100)	100 (80–100)	100 (83–100)	100 (78–100)
VMD	0 (0–8)	0 (0–35)	0 (0–55)	0 (0–17)	0 (0–22)
MD	0 (0–10)	0 (0–15)	0 (0–24)	N.D.*	N.D.*

*N.D. = Not determined; unable to calculate value due to lack of sensitive isolates within this sub-group
